# Supplementary material for: A sense of commitment to activity on Facebook: Evidence from a web-based paradigm
Source: PLoS One. 2023 Apr 18;18(4):e0271798. doi: 10.1371/journal.pone.0271798 (PMC10112986; doi:10.1371/journal.pone.0271798)
Supplement: Attachment — Submitted filename: reviewers comments.pdf [file pone.0271798.s001.pdf]

Reviewer comments

13th May 2022

Dear Author,

The topic of the research is actual and interesting. The development of four different experiments allows to investigate thoroughly the topic.

Methods and results are rigorous and well-described. The statistical analysis is adeguate for the number of observations recorded.

**Remarks**	
I suggest to merge the paragraphs (with relative sub-paragraphs) named:<br>2.1 Method, 3.1 Method, 4.1 Method, 5.1 Method<br>I suggest to merge even the paragraphs named:<br>2.2 Results, 3.2 Results, 4.2 Results, 5.2 Results<br><br>I understand you decided to maintain the four experiments divided, but it is very confusing. I suggest to create a unique section named 'Method' with a description of each experiment in sub-paragraphs. Evenly I suggest to create a unique section 'Results' with the four descriptions of results divided in sub-paragraphs.	
Please add a legend to the figures	
Please for Table 1 add some informations in the legend (for example the meaning of p value reported in parenthesis)	

Best regards
